# AHEAD Study: an observational study of the management of anticoagulated patients who suffer head injury

**DOI:** 10.1136/bmjopen-2016-014324

**Published:** 2017-01-13

**Authors:** Suzanne Mason, Maxine Kuczawski, M Dawn Teare, Matt Stevenson, Steve Goodacre, Shammi Ramlakhan, Francis Morris, Joanne Rothwell

**Affiliations:** 1 School of Health and Related Research, University of Sheffield, Sheffield, UK; 2 Emergency Department, Northern General Hospital, Sheffield, UK

**Keywords:** Warfarin

## Abstract

**Objectives:**

Management of anticoagulated patients after head injury is unclear due to lack of robust evidence. This study aimed to determine the adverse outcome rate in these patients and identify risk factors associated with poor outcome.

**Design:**

Multicentre, observational study using routine patient records.

**Setting:**

33 emergency departments in England and Scotland.

**Participants:**

3566 adults (aged ≥16 years) who had suffered blunt head injury and were currently taking warfarin.

**Main outcome measures:**

Primary outcome measure was rate of adverse outcome defined as death or neurosurgery following initial injury, clinically significant CT scan finding or reattendance with related complication within 10 weeks of initial hospital attendance. Secondary objectives included identifying risk factors for adverse outcome using univariable and multivariable analyses.

**Results:**

Clinical data available for 3534/3566 patients (99.1%), median age 79 years; mean initial international normalised ratio (INR) 2.67 (SD 1.34); 81.2% Glasgow Coma Scale (GCS) 15: 59.8% received a CT scan with significant head injury-related finding in 5.4% (n=208); 0.5% underwent neurosurgery; 1.2% patients suffered a head injury-related death. Overall adverse outcome rate was 5.9% (95% CI 5.2% to 6.7%). Patients with GCS=15 and no associated symptoms had lowest risk of adverse outcome (risk 2.7%; 95% CI 2.1 to 3.6). Patients with GCS=15 multivariable analysis (using imputation) found risk of adverse outcome to increase when reporting at least one associated symptom: vomiting (relative risk (RR) 1.8; 95% CI 1.0 to 3.4), amnesia (RR 3.5; 95% CI 2.1 to 5.7), headache (RR 1.3; 95% CI 0.8 to 2.2), loss of consciousness (RR 1.75; 95% CI 1.0 to 3.0). INR measurement did not predict adverse outcome in patients with GCS=15 (RR 1.1; 95% CI 1.0 to 1.2).

**Conclusions:**

In alert warfarinised patients following head injury, the presence of symptoms is associated with greater risk of adverse outcome. Those with GCS=15 and no symptoms are a substantial group and have a low risk of adverse outcome.

**Trial registration number:**

NCT02461498.

**Strengths and limitations of this study**

- This study is the largest to date that has identified and followed up the outcomes of 3534 patients taking warfarin who suffer head injury.
- Routinely available data from patient records were used thus missing data in some variables ranges from 9% to 42%. Owing to the known issues with using routine medical data, a strategy was employed to improve accuracy and minimise inconsistencies, as well as follow-up of missing data with hospital sites up to 10 weeks following initial hospital attendance.
- Missing data issues were handled by using multiple imputation in order to undertake the analysis for risk factors.

## Background

With over one million attendances reported in the UK and the USA annually, head injury is one of the most common injuries presenting to the emergency department (ED).^1–4^ Furthermore, up to 2.4% of the adult population of England per year are reportedly taking anticoagulation therapy,^5^ of which, warfarin is currently the most widely prescribed. These patients tend to be elderly and have comorbidities increasing their risk of falls and subsequent head injury. The management of anticoagulated patients following head injury therefore presents a substantial clinical challenge in an expanding and important group of patients.

Prior to January 2014, head injury guidance from the UK National Institute for Health and Care Excellence (NICE) did not specifically focus on managing patients receiving anticoagulation^6^ and current practice throughout England in the management of these patients varies considerably.^7^ This is also reflected in international guidelines for head injury produced in Scotland,^8^ Canada^9^ and USA,^10^ among others,^11–14^ where there is variation, largely due to the lack of a substantive evidence base to guide best practice. The uncertainty regarding the appropriate management of anticoagulated patients following an injury to the head, particularly relates to the use of CT,^15–21^ the value of measuring the international normalised ratio (INR),^22^ ^23^ and the need for hospital admission.^18^ ^21^ ^24^ To date there has been one adequately powered study of this group of patients,^17^ thus the risk of serious intracranial bleeding, adverse neurological outcome and death is uncertain. Previous studies of anticoagulated patients with head injury have identified the risk of subsequent intracranial bleeding to be between 5.1% and 7.8%,^17^ ^25^ ^26^ with other studies calculating an OR of between 2.73 and 5.48 for the same outcome compared with non-anticoagulated patients.^16^ ^27^ All of these studies also demonstrated wide variation in the investigation, admission and subsequent management of anticoagulation for these patients.

## Methods

### Setting and participants

We undertook an observational study across 33 hospital sites in England and Scotland. Adults (≥16 years) attending the ED in a participating hospital site between September 2011 and March 2013 presenting with head trauma who were currently taking warfarin were included.

We defined head trauma as any non-penetrating head injury above the neck irrespective of mechanism.^28^ Patients experiencing multisystem trauma were included in the study. We excluded patients with a penetrating injury or head trauma following a spontaneous intracranial event.

### Data collection

Research staff within the hospital sites identified consecutive patients from all attendances at the respective ED and recorded basic demographic information, attendance details, injury mechanism and clinical examination data from using routinely available medical records. The latter included initial documented Glasgow Coma Scale (GCS), other physiological observations, symptoms and evidence of trauma, and results of any investigations, all collected via a standardised study web-based data form. Investigations were undertaken according to perceived clinical need and no additional investigations were mandated as part of the study. To minimise missing data, inconsistencies and improve accuracy, a strategy was employed for reviewing patient medical records (see online supplementary table S1),^29^ as well as follow-up with research staff up to 10 weeks after initial attendance. CT scan reports were retrospectively reviewed by an independent expert clinical working group and a preagreed classification assigned to the findings. The expert clinical working group were five emergency medicine consultants who had access to same information as ED clinicians at the hospital site (the investigative data—observation and blood results) in order to facilitate classifying any abnormalities reported on the CT scan. The classification (table 1) was developed specifically for the study and agreed by the expert working group and the study steering committee, prior to any reviewing.

**Table 1:** CT scan classification

Classification	Description
1	Intracranial abnormality likely to be due to injury (eg, subdural, extradural, contusion, etc)
2	Other abnormality likely to be due to injury (eg, scalp haematoma, uncomplicated fracture, etc)
3	Other abnormality unlikely to be due to injury
4	Normal CT scan

10.1136/bmjopen-2016-014324.supp1 supplementary table Strategy employed to minimise missing data and inconsistencies, and improve accuracy of AHEAD data collection, using routine ED patient records (recommended by Gilbert et al, 1996).[29]

Based on 381 CT scans reviewed by five reviewers—Krippendorff's α=0.816 (95% CI 0.765 to 0.862) suggesting good degree of reliability.^30^

Every effort was made to identify consecutive eligible patients in order to minimise missing eligible patients through reviewing patient attendances with head injury, those taking warfarin, and also by checking which patients received a head CT, or had their INR checked.

Ethical approval for the study was obtained and an ‘opt-out’ method was adopted where patients were informed of their inclusion in the study on receipt of a study pack containing information about the study and how to ‘opt-out’. This was mailed to the patient's home address 6 weeks after attendance. Patients identified as still being admitted to the hospital at this point were contacted directly by the hospital research nurse.

The study aimed to determine the rate of adverse outcome associated with head injury. The primary outcome of interest was the rate of adverse outcome defined by death or neurosurgery resulting from the initial injury, a clinically significant CT scan finding (classification 1 from table 1) or reattendance to the hospital with a significant head injury-related complication up to 10 weeks after the original attendance. Identifying risk factors for adverse outcome was a secondary objective.

### Sample size

The study was powered to detect a clinically important relative risk of 2 for up to 10 potential clinical risk factors. Assuming the population risk is 5%, 3000 patients would result in 150 cases. This number of cases (and the same number of controls) would correspond to 80% power at the (Bonferroni corrected) 0.5% level to detect a risk factor with a 20% frequency in controls. Assuming the true risk is 5%, the sample size of 3000 would give a precise estimate of the population risk where the expected 95% CI would have a width of 0.016.

### Statistical methods and data analyses

All analyses were conducted using Stata V.13. The study was a closed cohort design and hence risks and relative risks could be reported. Clustering within the 33 EDs was allowed for in the analysis by using multilevel Poisson regression with robust SE estimation. All reported relative risks and 95% CIs have been adjusted for the clustering by ED. Non-comparative proportions and risks and their 95% CIs are reported without adjusting for clustering. The primary outcome for the statistical analysis was an adverse outcome related to the head injury.

At the study planning stage we set a Bonferroni corrected significance threshold of 0.005 to allow for the multiple testing for up to 10 risk factors. However, rather than making this a formal adjustment we have reported the nominal p values and unadjusted 95% CIs. We have considered GCS as a categorical variable with four levels (GCS=15, GCS=14, GCS=13, GCS<13), INR as both a numerical and binary variable, and four binary neurological symptoms.

Multiple imputation for missing data was performed using the Realcom software (http://www.bristol.ac.uk/cmm/software/realcom/). This software supports multiple imputation using chained equations and allows for multilevel or clustered data. The variables included in the multiple imputation (which was limited to participants with GCS=15) were adverse outcome (primary outcome), age, gender, log(INR), the four neurological symptoms (headache, vomiting, amnesia and loss of consciousness; secondary outcomes) and the hospital ED. This generated 100 imputed data sets which were then analysed in Stata V.13 using Rubin's combination rules to form one set of results.^31^

## Results

Over the 19-month period, 3566 patients were enrolled in the study excluding 154 patients that requested they be withdrawn. Anonymised clinical data were submitted for nearly all patients (99%, n=3534).

Of the 3534 included patients, the age range was 18–101 years (median 79 years; IQR=12) with the majority arriving by ambulance (73.8%, n=2607) and presenting following a fall (91.6%, n=3238). The most common presenting diagnosis recorded in 91.4% (n=3229) was head wound (table 2).

**Table 2:** Patient demographics

	All patients	Missing data
	n (%)	N (%)
Total	3534	
Gender		0
Males	1738 (49.2)	
Age group, years		0
<60	251 (7.1)	
60–69	313 (8.9)	
70–79	925 (26.2)	
80–89	1674 (47.4)	
90+	371 (10.5)	
Symptoms, type		
Amnesia	341 (9.6)	1464 (41.4)
Vomiting	163 (4.6)	900 (25.5)
Loss of consciousness	425 (12.0)	620 (17.5)
Headache	535 (15.1)	1511 (42.8)
Number of symptoms		0
0	2428 (68.7)	
1	824 (23.3)	
2+	282 (8.0)	
Admitted		0
Yes	2216 (62.7)	
Length of stay, days		0
0	341 (9.6)	
1–2	975 (27.6)	
3–10	413 (11.7)	
11+	487 (13.8)	
Glasgow Coma Scale		0
15	2871 (81.2)	
14	275 (7.8)	
13	23 (0.7)	
<13	60 (1.7)	
Not recorded at site	305 (8.6)	
INR		78 (2.2)
<2	741 (21.0)	
2–4	1941 (54.9)	
>4	252 (7.1)	
Not performed at site	522 (14.8)	
CT scan performed		0
Yes	2114 (59.8)	
Time to scan (from ED attendance)		195 (5.5)
<1 hour	199 (9.4)	
1–4 hours	1210 (57.2)	
4+ hours	610 (28.9)	
CT grading		135 (3.8)
Intracranial abnormality likely to be due to injury	192 (5.4)	
Other abnormality likely to be due to injury (eg, scalp haematoma, uncomplicated fracture)	417 (11.8)	
Other abnormality unlikely to be due to injury	909 (25.7)	
Normal CT scan	461 (13.0)	
Reversal therapy		179 (5.1)
Yes	189 (5.3)	
Prothrombin complex	30 (0.8)	
Intravenous vitamin K	100 (2.8)	
Oral vitamin K	16 (0.5)	
Other*	42 (1.2)	
Neurosurgical procedures		36 (1.0)
Yes	18 (0.5)	
Further hospital attendances		0
Head injury-related to original attendance	37 (1.0)	
Died		0
Yes	249 (7.0)	
Head injury-related	41 (1.2)	
Other	158 (4.5)	
Not known	50 (1.4)	
Overall adverse outcome rate	208 (5.9)	

*Included combinations of reversal therapy given (prothrombin complex+vitamin K=38; prothrombin complex+vitamin K+platelets+tranexamic acid=1; fresh frozen plasma+vitamin K+platelets=1) and vitamin K (intravenous or oral not known)=1.

ED, emergency department; INR, international normalised ratio.

Over two-thirds (68.7%, n=2428) of patients did not have any associated head injury symptoms reported (amnesia, vomiting, loss of consciousness or headache). On initial evaluation in the ED, 81.2% (n=2871) patients had a GCS score of 15 and 60 (1.7%) patients had a GCS of 12 or lower, indicating moderate-to-severe head injury. INR was measured in 83% (n=2934) of patients and the median value was 2.4 (IQR=1.9–3.0), with less than one-third of patients having a measurement outside of the normal therapeutic range (INR=2–4)^32^ (INR<2: 21.0%, n=741; INR>4: 7.1%, n=252). Overall, 59.8% of patients (n=2114) received a CT scan which was consistent with a classification 1 complication in 5.4% (n=192).

Other adverse outcomes included neurosurgery in 0.5% (n=18) patients, a related head injury reattendance in 1.0% (n=37), and a head injury-related death in 1.2% (n=41). This produced an overall adverse outcome rate for the whole cohort of 5.9% (n=208, 95% CI 5.2 to 6.7). The adverse outcome rate included patients only once irrespective of whether they experienced multiple adverse outcomes.

### Risk factors for adverse outcome

The variables considered as potential risk factors in the univariable analysis were GCS, INR, vomiting, amnesia, loss of consciousness and headache with age and sex as potential confounders. The aim of this analysis was to identify predictors of adverse outcome to assist in clinical decision-making. All of these variables (except for age and sex) were found to be statistically significant at the 5% level in a univariable analysis.

### Glasgow Coma Scale

GCS was recorded for 3229 patients (91.4%). While GCS was the strongest predictor of risk, we found patients presented with a GCS below 15 rarely (11.1%, n=358). We therefore considered this risk factor alone. The lowest risk is for those with GCS=15 (table 3), with GCS<15 being a strong risk factor. Three hundred and five patients did not have a recorded GCS, although their risk of adverse outcome was lower and not significantly different to the GCS=15 group.

**Table 3:** Univariable analysis of GCS

GCS value	Patients n	Adverse outcome n (%)	Relative risk * (compared with GCS=15)	95% CI*	p Value
15	2871	124 (4.3)	1	NA	
14	275	37 (13.4)	3.11	2.20 to 4.41	<0.001
13	23	9 (39.1)	8.79	5.37 to 14.37	<0.001
12 and below	60	29 (48.3)	10.53	7.90 to 15.36	<0.001
Below 15	358	75 (20.9)	4.82	3.66 to 6.35	<0.001
GCS missing	305	9 (3.0)	0.65	0.34 to 1.39	0.296

*Relative risks and 95% CIs estimated using multilevel Poisson regression to allow for clustering by hospital site.

GCS, Glasgow Coma Scale; NA, not available.

### International normalised ratio

**Figure 1:**
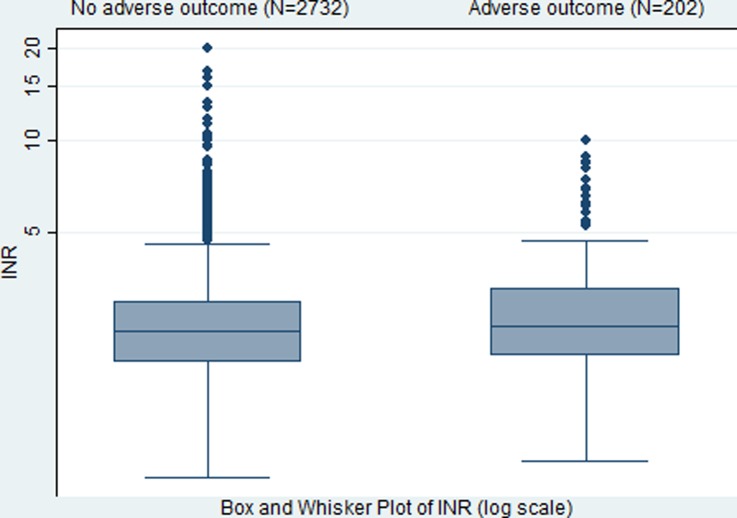
Box and Whisker plot of log(INR) by adverse outcome. INR, international normalised ratio.

INR was recorded in 2934 patients (n=522 not performed at site and n=78 missing). The median INR in those with an adverse outcome is slightly higher than those without an adverse outcome (2.5 vs 2.4; figure 1).

Univariable Poisson regression found the continuous variable INR was statistically significantly positively associated with a higher risk of adverse outcome (p=0.029). However, this association reduced (relative risk=1.11, 95% CI 0.95 to 1.18, p=0.298) when patients with GCS below 15 were excluded. The risk of adverse outcome in those 600 patients with INR missing was 2.0% (95% CI 1.14% to 3.49%).

### Neurological symptoms

We considered each of the neurological symptoms: amnesia; vomiting; headache and loss of consciousness in all patients, and then in patients with GCS=15 only (table 4). There were missing values for each of the symptoms (table 2).

**Table 4:** Univariable analysis results grouped by neurological symptoms category

Symptom	Patients	Non-missing, n	Risk in those symptom positive, % (95% CI)	Risk in those symptom negative, % (95% CI)	Relative risk*	95% CI	p Value
Vomiting	All	2634	15.95 (9.51 to 9.67)	4.05 (3.34 to 4.90)	3.94	2.32 to 6.70	<0.001
	GCS=15 only	2237	9.84 (5.65 to 16.56)	3.26 (2.58 to 4.11)	3.00	1.68 to 5.41	0.001
Amnesia	All	2070	14.96 (11.54 to 19.16)	3.47 (2.70 to 4.43)	4.37	3.05 to 6.25	<0.001
	GCS=15 only	1796	14.07 (10.36 to 18.83)	2.87 (2.14 to 3.84)	4.90	3.34 to 7.19	<0.001
Headache	All	2023	7.66 (5.69 to 10.25)	3.63 (2.79 to 4.71)	2.11	1.33 to 3.34	0.001
	GCS=15 only	1723	5.64 (3.87 to 8.15)	3.17 (2.33 to 4.30)	1.78	0.97 to 3.26	0.062
LOC†	All	2914	14.82 (11.75 to 18.53)	3.58 (2.91 to 4.38)	4.14	2.92 to 5.88	<0.001
	GCS=15 only	2475	10.48 (7.61 to 14.26)	2.99 (2.35 to 3.80)	3.50	2.26 to 5.41	<0.001

*Compared with no symptoms.

†LOC=loss of consciousness.

GCS, Glasgow Coma Scale.

Patients with GCS=15 and no reported symptoms accounted for a significant proportion of the cohort (55.8%, n=1973) and had the lowest risk of an adverse outcome (risk 2.7%, 95% CI 2.1 to 3.6). The group of 1973 patients where no symptom was reported as present included a substantial number of patients where at least one symptom report was missing or not recorded (n=1171). In those patients with no missing data for symptoms, this risk was further reduced (n=802, risk 2.1%, 95% CI 1.3 to 3.4). Each of the symptom variables was statistically significantly associated with increased risk of an adverse outcome. With the exception of the symptom headache, the associations remained statistically significant after the exclusion of patients with GCS below 15.

In univariable analyses for each symptom the risk of an adverse outcome was statistically significantly raised when the symptom was missing (compared with those with no symptom present). In general, single symptoms were more likely to be missing if there was at least one positive symptom reported. The patterns of missing data suggest that an analysis limited to the complete records may not be representative of the full cohort and we may obtain biased results when attempting to fit multivariable models. We therefore used multiple imputation to impute values for the four neurological symptoms in those patients with GCS=15.

The univariable analysis shows a similar pattern to that found in table 4 for those with GCS=15. However, following the imputation the symptom headache is now statistically significant at the 5% level. The multiple imputation permitted a full multivariable model to be fitted to examine joint associations (table 5). When all four symptoms are included in the same model amnesia is the strongest predictor with vomiting or loss of consciousness associated with slightly lower relative risks and headache associated with the lowest relative risk. It should be noted that the baseline reference group in the joint analysis is the group of patients with no symptoms reported. In the joint analysis only two of these symptoms are statistically significant; however, all the 95% CIs include the relative risk of 2 suggesting that all four symptoms may have important clinical significance. The analysis following multiple imputation assumes that all these neurological symptoms are measurable which may not be the case (eg, headache is subjective). However, the analysis following imputation provides a means to assess how the presence of up to four symptoms contributes to overall risk.

**Table 5:** Relative risk in patients GCS=15 associated with neurological symptoms following multiple imputation (n=2871)

Neurological symptom	Relative risk*	95% CI	p Value
Univariable analysis			
Amnesia	4.83	3.22 to 7.23	<0.001
LOC†	3.49	2.30 to 9.95	<0.001
Vomiting	3.00	1.66 to 5.24	<0.001
Headache	1.75	1.04 to 2.84	0.016
Multivariable joint analysis			
Amnesia	3.48	2.13 to 5.70	<0.001
Vomiting	1.80	0.97 to 3.36	0.063
LOC†	1.75	1.03 to 2.99	0.039
Headache	1.30	0.76 to 2.22	0.331

*Compared with no symptoms.

†LOC=loss of consciousness.

### Missing data

Missing data have been considered throughout the statistical analysis, examining the risk in those with missing data on a variable by variable basis. The missing data in the reporting of neurological symptoms was clearly an important issue, with headache and amnesia being most commonly missing. These are symptoms that would be more difficult for an observer to report than a patient. There may be good clinical reason why some symptoms cannot be reported such as older patients with pre-existing memory problems not being able to report amnesia. It is of some concern that around one-third of the data cannot be assessed for presence of a neurological symptom. Hence, we cannot be confident that data are missing at random. Assuming that the data are missing at random we have used a multiple imputation approach to allow us to examine how the risk factors may act together.

## Discussion

The overall risk of adverse outcome in the cohort was 5.9%. The study has shown that patients with a GCS of 15 accounted for a significant proportion of the study cohort (88.9%) and that in those with no associated neurological symptoms, the risk of adverse outcome is low (2.7%), with risk increasing as neurological symptoms increase and GCS falls (see box 1). The multivariable analysis found that in patients with GCS=15, while all four neurological symptoms are important in terms of increasing the risk of adverse outcome, only amnesia and loss of consciousness reached statistical significance. INR, a controversial measurement often used as a guide in the management of patients' care, was found to show no association with adverse outcome once other risk factors are included.

Box 1 **Adverse event rate by Glasgow Coma Scale (GCS) and neurological symptoms**

- GCS=15 and no neurological symptoms (n=2243): adverse event=2.8% (n=65)
- GCS=15 and one neurological symptom (n=384): adverse event=9.0% (n=38)
- GCS=15 and two neurological symptoms (n=109): adverse event=13.5% (n=17)
- GCS=15 and three neurological symptoms (n=15): adverse event=26.7% (n=4)
- GCS<15 (n=358): adverse event=20.9% (n=75)

This study is the largest of its kind with sufficient power to describe the outcomes of a cohort of anticoagulated head injury patients presenting to the ED, and their predictors for an adverse outcome. The adverse outcomes we have described are comparable with those presented in some previous studies that also report on complication rates for anticoagulated patients separately.^17^ ^25^ However, other studies have reported much higher incidences of complications among this population.^16^ ^21^ ^26^ ^33^ ^34^ This is largely down to the previous studies either being inadequately powered with smaller study sizes (cohorts range from 32 to 1064 included patients), from single site studies, or a study that includes all minor head injury regardless of anticoagulation status with subgroup analysis of anticoagulated patients.

The majority of international guidance on the management of head injury does not advise specifically on the care of patients who are anticoagulated mainly due to the lack of sufficiently powered studies to address management in such a subpopulation.^9^ ^12^ Guidance from NICE^2^ ^6^ has changed based on the review of a number of studies judged by NICE to be of low quality. As a result, the current guidance recommends a CT scan for all anticoagulated patients within 8 hours of suffering a head injury regardless of the presence of any other indication for a scan. This would significantly increase workload and costs for hospitals. Equally the National Emergency X-Radiology Utilisation Study (NEXUS II), CT in Head Injury Patients (CHIP), American College of Emergency Physicians (ACEP) head CT and the European Federation of Neurological Societies (EFNS) advocate that all patients taking warfarin should have an immediate CT scan irrespective of injury severity, GCS or neurological symptoms.^10–13^ Guidance from SIGN recommends admission to hospital for these patients, but interestingly, not a CT scan.^8^ It is unclear what evidence this guidance is based on. Guidance for the management of non-anticoagulated head injuries has demonstrated the value of including clinical features when deciding whether to investigate patients.

This study has shown that (1) head injury symptoms and GCS can be used to predict adverse outcome in anticoagulated patients suffering blunt head trauma, (2) INR does not predict adverse outcome in those patients with GCS=15, (3) patients with GCS=15 and no symptoms have a low risk of adverse outcome regardless of INR (2.7%). Therefore, use of CT scanning in low-risk patients may be of limited value, but the decision to recommend CT scanning in guidance should take into account the potential benefits, harms and costs of CT scanning. Furthermore, our estimate of the low risk of adverse outcome in those with GCS=15 and no symptoms needs to be confirmed in other cohorts.

Further research is therefore needed to validate our findings on a separate cohort of anticoagulated patients, while decision analysis modelling is required to compare the potential benefits, harms and costs of CT scanning in low-risk patients. In addition, further work is needed on the newer oral anticoagulants and antiplatelet drugs in order to inform clinical practice.

## Limitations

The study was limited by not having a gold standard reference test for adverse outcome. For pragmatic reasons, we undertook this observational study applying a range of adverse outcomes. It is possible that a small number of adverse outcomes would have been missed, although every effort was made by the study team to ensure this did not happen. Patients with an adverse outcome may have been missed if they had died in the community or attended another hospital with a delayed complication thereby underestimating the proportion of adverse outcomes in the study. The data collection process was developed locally to suit each service model and as such, the study was partially compromised by having some data items missing. A strategy was employed throughout the study to try to minimise missing data and improve accuracy, as well as undertaking follow-up with each hospital site up to 10 weeks after patient attendance as recommended by Gilbert *et al*,^29^ when using medical records. The missing items mainly included recording the symptoms of amnesia and headache which we found were far less likely to be documented than the symptoms of vomiting and loss of consciousness. It is likely that clinicians were less inclined to record amnesia and headache as these are symptoms that cannot readily be observed, and can be subject to uncertainty especially in older patients with cognitive impairment. However, our analysis included an extensive missing data analysis which increased our confidence in the study findings.
