# Supplementary material for: AHEAD Study: an observational study of the management of anticoagulated patients who suffer head injury
Source: BMJ Open. 2017 Jan 13;7(1):e014324. doi: 10.1136/bmjopen-2016-014324 (PMC5253554; doi:10.1136/bmjopen-2016-014324)
Supplement: supplementary table — Strategy employed to minimise missing data and inconsistencies, and improve accuracy of AHEAD data collection, using routine ED patient records (recommended by Gilbert et al, 1996).[29] [file bmjopen-2016-014324supp_table.pdf]

Supplementary Table 1: Strategy employed to minimise missing data and inconsistencies, and improve accuracy of AHEAD data collection, using routine ED patient records (recommended by Gilbert et al, 1996).[29]

Training	Prior to study commencement, all research staff at each participating hospital site were trained in abstracting the appropriate data from patient ED medical records. The research staff were requested to practise using the study web-based data form by submitting 2 'practice' records. This process was repeated for any new research staff joining the study at a later date.
Case selection	Explicit protocol was issued to each participating hospital site which described the inclusion and exclusion criteria for the study. The study web-based data form also included a question to check patients' eligibility to the study.
Definition of variables	A study Dataset Manual was issued to each participating hospital site, defining all of the variables on the study web-based data form that needed to be collected.
Abstraction forms	The study web-based data form was used by research staff at each participating hospital site in conjunction with a Dataset Manual and web-based data form Guidelines. The web-based data form was only accessible to research staff after completing one-to-one training by a member of the AHEAD study team.
Meetings	Regular contact to all participating hospital sites was undertaken by email, providing feedback on patient recruitment on a monthly basis.
Monitoring	The AHEAD study team regularly ran reports to review the amount and quality of data submitted to the study web-based data form. Any issues identified were highlighted to the research staff at the participating hospital site and followed-up by telephone as appropriate.
Blinding	Blinding research staff to the purpose of the AHEAD Study was not undertaken.
Testing of interrater agreement	22 of the 33 (67%) participating hospital sites were visited by a member of the AHEAD study team and up to 6 patient records were re-abstracted. These records were re-submitted to the study web-based data form, with the second reviewer blinded to the original data submission. The measure of agreement found on this re-abstraction ranged from 0.19 to 2.88%.
